# Printing and Rerouting of Elastic and Protease Responsive Shape Memory Hydrogel Filaments

**DOI:** 10.1002/adhm.202502262

**Published:** 2025-06-20

**Authors:** Philip Lifwergren, Viktoria Schoen, Sajjad Naeimipour, Lalit Khare, Anna Wunder, Hanna Blom, Jose G. Martinez, Pierfrancesco Pagella, Anders Fridberger, Johan Junker, Daniel Aili

**Affiliations:** ^1^ Laboratory of Molecular Materials Division of Biophysics and Bioengineering Department of Physics, Chemistry, and Biology Linköping University Linköping 583 81 Sweden; ^2^ Sensor and Actuator Systems Department of Physics, Chemistry and Biology Linköping University Linköping 581 83 Sweden; ^3^ Department of Biomedical and Clinical Sciences Linköping University Linköping 581 85 Sweden; ^4^ Center for Disaster Medicine and Traumatology Department of Biomedical and Clinical Sciences Linköping University Linköping 581 85 Sweden

**Keywords:** 3D bioprinting, filament, hydrogel, REFRESH, tubular structures

## Abstract

The fabrication of mechanically robust and reconfigurable hydrogel filaments remains a major challenge in biofabrication of perfusable architectures, dynamic tissue models, and complex 3D cell‐laden constructs. Conventional extrusion‐based bioprinting techniques generate filaments that are soft and fragile, limiting post‐processing, scalability, and functional adaptability. Rerouting of Free‐Floating Suspended Hydrogel Filaments (REFRESH) is introduced as a biofabrication strategy that integrates an aqueous two‐phase system (ATPS)‐compatible elastic extracellular matrix mimicking bioink material with a flexible printing and post‐processing approach to overcome these constraints. This method enables the formation of highly elastic hydrogel filaments cross‐linked via strain‐promoted azide‐alkyne cycloaddition (SPAAC) of bicyclo[6.1.0]non‐4‐yne‐functionalized hyaluronan, exhibiting a strain at break exceeding 100%. The printed filaments maintain mechanical integrity during manual handling and post‐processing using textile‐inspired techniques, such as knotting and braiding, into reconfigurable 3D architectures. A distinct shape memory function enables programmed mechanical actuation and recovery of deformed structures. The hydrogel system supports high cell viability across multiple cell types and enables the fabrication of multicellular constructs with spatially defined organization. By incorporating protease‐degradable cross‐linkers, REFRESH‐generated filaments function as sacrificial templates for perfusable tubular structures. This approach significantly expands the biofabrication design space, offering new possibilities for engineering vascularized tissues and complex hydrogel‐based architectures.

## Graphical abstract

A biofabrication strategy, Rerouting of Free‐Floating Suspended Hydrogel Filaments (REFRESH), is developed enabling the printing of highly elastic, reconfigurable protease‐responsive hydrogel filaments with shape memory properties. The filaments can be manually braided, knotted, and rerouted to create intricate cell‐laden 3D architectures, expanding the design space for engineered tissues and biomimetic hydrogel systems.

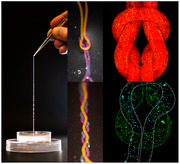

## 1 Introduction

The development of advanced biofabrication techniques for processing cell‐laden, extracellular matrix (ECM)‐mimetic hydrogels, including 3D bioprinting, has significantly expanded the potential for engineering complex tissue constructs, physiologically relevant disease models, and functional, transplantable tissues.^[^ ^1^, ^2^, ^3^, ^4^ ^]^ However, despite these advances, a persistent challenge remains in the fabrication of scalable, mechanically robust, and physiologically functional hydrogel‐based architectures, including filamentous and tubular structures, for engineering of structurally intricate 3D cell‐laden constructs^[^ ^5^ ^]^ and systems requiring perfusable vascularization.^[^ ^6^, ^7^, ^8^, ^9^ ^]^ Non‐tubular and tubular filamentous hydrogel structures can be obtained by a range of different techniques based on wet spinning or extrusion bioprinting using viscous non‐cross‐linked bioinks or shear‐thinning hydrogels.^[^ ^5^, ^6^, ^7^, ^10^, ^11^ ^]^ Although conventional extrusion‐based bioprinting is extremely versatile for generating cell‐laden 3D objects,^[^ ^6^ ^]^ especially in combination with embedded bioprinting techniques,^[^ ^12^, ^13^, ^14^, ^15^, ^16^, ^17^, ^18^ ^]^ creating topologically more complex and intertwined 3D structures requires more sophisticated methods.^[^ ^19^ ^]^ Moreover, the resulting hydrogel filaments obtained after extrusion bioprinting are inherently soft and mechanically fragile, posing significant challenges in handling, post‐processing, and maintaining structural integrity, thereby limiting their translational potential.

Aqueous two‐phase systems (ATPS) have emerged as a promising technique for filament fabrication, leveraging interfacial stabilization between immiscible aqueous polymer solutions to enable the controlled deposition of soft hydrogel structures.^[^ ^20^, ^21^, ^22^, ^23^ ^]^ While ATPS allows for controlled phase separation and biofabrication of distinct polymer‐rich filaments, its use has been constrained by a limited selection of ATPS compatible bioink materials, restricting the range of achievable mechanical and biochemical properties. Additionally, ATPS‐generated filaments often lack the mechanical robustness and shape stability required for post‐processing or direct integration into large‐scale, architecturally intricate biofabricated structures.

**Figure 1:**
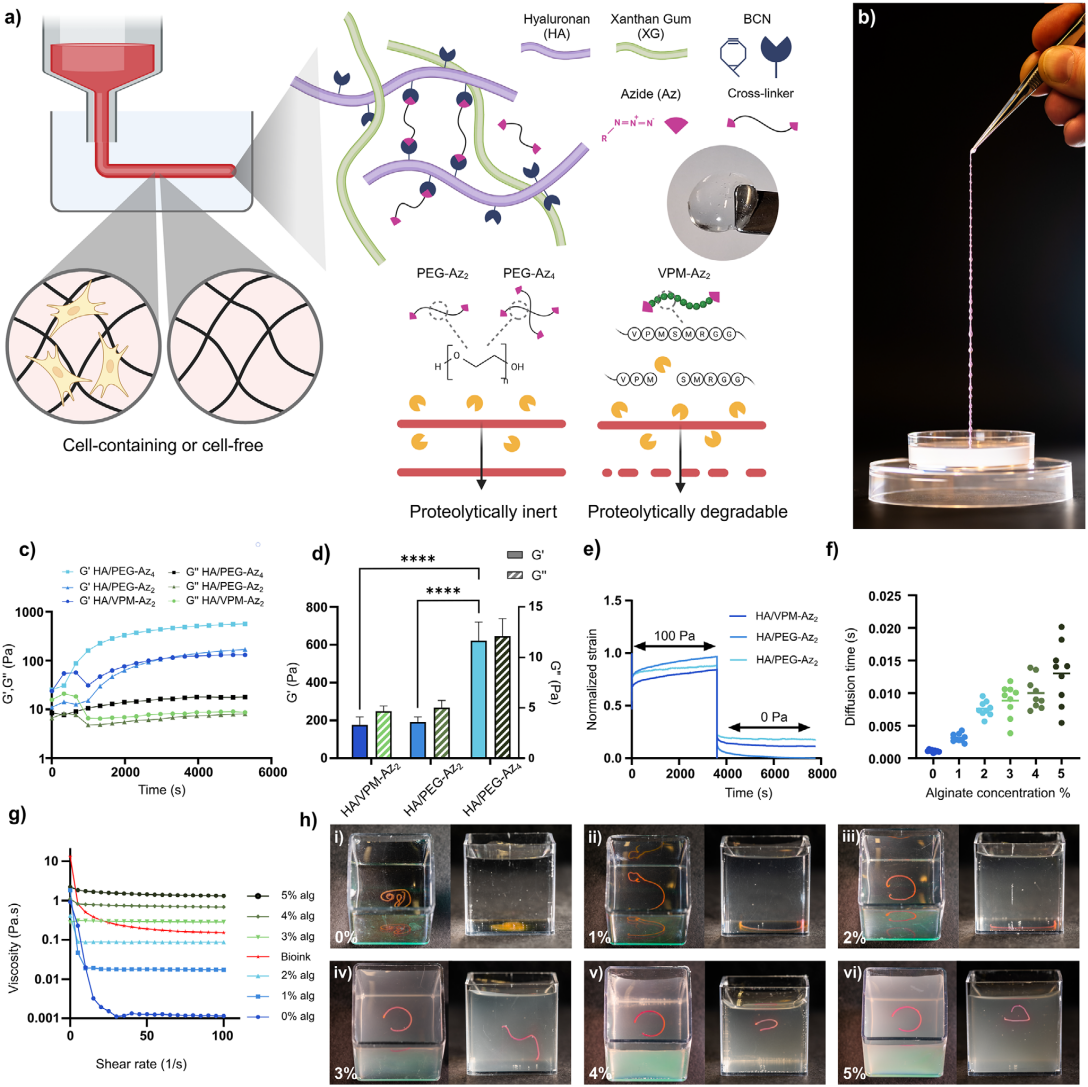
a) Schematic illustration of the HA/PEG hydrogel system. b) Photograph of a fully hydrated hydrogel filament extracted after REFRESH printing, decorated with water droplets. c) Hydrogel gelation kinetics. d) Storage (G′) and loss (G″) modulus of the hydrogels after full cross‐linking. e) Creep‐recovery test. f) Diffusion time of Cy5‐Az‐labeled HA‐BCN in HEPES buffer (0% alginate) or in alginate solutions with concentrations from 1–5% (w/v). g) Dynamic viscosity of buffer (0% alginate), 1–5% (w/v) alginate and bioink. h) Photos of HA/PEG filaments printed in buffer (0% alginate) i) or 1–5% (w/v) alginate ii‐vi). Top (left) and side (right) view.

Here, we introduce Rerouting of Free‐Floating Suspended Hydrogel Filaments (REFRESH) as a novel biofabrication strategy that integrates ATPS‐compatible and highly elastic extracellular matrix (ECM) mimicking bioink materials with an advanced printing and post‐processing approach to create hydrogel filament structures that overcome the mechanical and geometrical limitations of conventional extrusion bioprinting. Unlike most conventional suspended hydrogel filaments, REFRESH‐generated filaments exhibit exceptional mechanical robustness, with a strain at break exceeding 100%, enabling them to be manually rerouted, knotted, braided, and reconfigured into intricate architectures without structural failure (**Figure** **1**a). The hydrogels, which are obtained by bioorthogonal cross‐linking using strain‐promoted azide‐alkyne cycloaddition of bicyclo[6.1.0]non‐4‐yne (BCN) functionalized hyaluronan, allow for 3D culture of a wide range of cell‐types for generating advanced tissue and disease models.^[^ ^24^, ^25^, ^26^, ^27^ ^]^ Cells encapsulated in the printed filaments demonstrated high viability, even after manual processing. The possibilities to postprocess hydrogel filaments using techniques adopted from, e.g., the textile industry could significantly expand the biofabrication design space and facilitate large‐scale production of complex hydrogel‐based structures. Moreover, the covalently cross‐linked REFRESH filaments have a distinct shape memory function that provides novel means for mechanical actuation of printed structures. Using protease‐responsive peptide‐based cross‐linkers, we show that the hydrogel filaments can also be employed as sacrificial elements for the fabrication of perfusable tubular structures with geometries that cannot be obtained using conventional extrusion bioprinting (e.g., knots) and using benign proteases for filament degradation. REFRESH significantly expands the potential of extrusion‐based bioprinting and facilitates the design of sophisticated and complex filamentous, tubular, and cell‐laden 3D constructs and could pave the way for implementation of novel advanced hydrogel filament postprocessing technologies.

## 2 Results and Discussion

### 2.1 REFRESH Printing of HA/PEG and HA/VPM Hydrogels

To enable 3D printing of elastic and cytocompatible robust ECM‐mimicking hydrogel filaments with defined dimensions, desired mechanical properties, and controlled proteolytic degradation, we modified hyaluronan with bicyclo[6.1.0]non‐4‐yn‐9‐ylmethyloxycarbonyl]‐1,8‐diamino‐3,6‐dioxaoctane (HA‐BCN) (Figure S1, Supporting Information), allowing for cross‐linking using azide‐terminated peptides and linear and multi‐arm poly(ethylene glycol) (PEG‐Az_n_, *n* = 2, 4) by strain‐promoted alkyne–azide cycloaddition (SPAAC). We utilized 2‐arm PEG‐Azide (PEG‐Az_2_), 4‐arm PEG‐Azide (PEG‐Az_4_), as well as a protease‐degradable peptide cross‐linker with terminal azide moieties (VPM‐Az_2_), resulting in HA/PEG‐Az_2_, HA/PEG‐Az_4_, and HA/VPM‐Az_2_ hydrogels, respectively (Figure 1a). Xanthan gum (XG) was included to improve the viscosity and printability of the bioink materials.^[^ ^24^, ^28^ ^]^ Cross‐linking of the hydrogels commenced immediately upon mixing HA‐BCN with the cross‐linkers. The hydrogels reached their final stiffness after approximately 2 hours after ATPS‐mediated printing, resulting in filaments that could be extracted using a tweezer (Figure 1b,c). Note that the apparent discontinuities and irregularities observed along the filament contour in the photograph in Figure 1b are due to surface‐adhered water droplets formed during handling and imaging, and do not reflect structural inhomogeneities or defects in the hydrogel filament. The hydrogels were viscoelastic with a storage modulus (G′) of 0.18 ± 0.04, 0.19 ± 0.03, and 0.62 ± 0.09 kPa for HA/VPM‐Az_2_, HA/PEG‐Az_2_, and HA/PEG‐Az_4_, respectively, when fully hydrated in buffer (pH 7.4) (Figure 1c,d; Figure S2, Supporting Information). The higher stiffness observed for HA/PEG‐Az_4_ hydrogels reflects the higher cross‐linking density achieved with the four‐arm cross‐linker, as compared to the linear PEG‐Az_2_ and VPM‐Az_2_ cross‐linkers. The hydrogels were highly elastic and demonstrated minimal plastic deformation when fully hydrated (Figure 1e).

We have previously demonstrated that HA/PEG‐Az_n_ (*n* = 2, 4, 8) hydrogels can serve as excellent bioink materials for extrusion 3D bioprinting and for generating tissue and disease models.^[^ ^24^, ^25^, ^27^ ^]^ Since the hydrogels are very soft immediately after printing, we have previously used a gelatin slurry as a temporary support, which enabled the printing of elaborate 3D architectures.^[^ ^25^ ^]^ However, the microstructure of the gelatin beads in the support bath resulted in a rough surface of the structures, which could be detrimental to the yield strength of thin bioprinted hydrogel filaments. To circumvent this issue, we investigated the possibilities to stabilize the hydrogels by ATPS during the cross‐linking of printed filaments using aqueous alginate solutions as a temporary support. To function as a support material, mixing between the alginate and the printed hydrogel components should ideally be negligible. The diffusion rate of HA‐BCN in alginate solutions was investigated using fluorescence correlation spectroscopy (FCS) and was found to decrease linearly with increasing alginate concentrations ranging from 1–5% (w/v) due to the increase in viscosity of the solution (Figure 1f). The dynamic viscosity of alginate in the shear rate interval of 10–100 1/s increased from 0.019 ± 0.008 Pa.s in 1% (w/v) alginate to 1.47 ± 0.17 Pa.s in 5% (w/v) alginate (Figure 1g). When extruding the filaments in 1–2% (w/v) alginate, the low viscosity of the solution resulted in turbulence and a tendency to form irregular filamentous structures. Additionally, the filaments became very fragile, likely due to inefficient ATPS stabilization causing dilution of the hydrogel components during the cross‐linking process. Printing in 4–5% (w/v) alginate typically resulted in short and clustered filaments due to drag forces resulting in high variability in filament thickness. In contrast, when matching the viscosity (0.29 ± 0.21 Pa.s) and the concentration (3% w/v) of the supporting alginate solution with the viscosity (0.22 ± 0.09 Pa.s) and concentration (2.8‐3.3% w/v) of the bioink material, defined ATPS stabilized filaments could be produced (Figure 1b,h). Whereas filaments printed in 1–2% (w/v) alginate had a tendency to sink to the bottom of the container, filaments printed in 3% (w/v) alginate remained suspended in the alginate solution due to the density matching between the alginate and the bioink. Filaments printed in 4–5% (w/v) alginate accumulated at the alginate/air interface due to the lower density of the filament compared to the alginate solution (Figure 1h).

**Figure 2:**
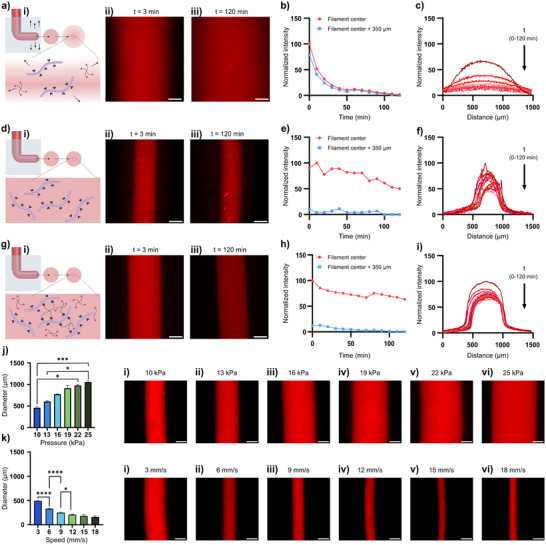
a) i) HA/PEG‐Az_4_ filament printed in buffer at ii) *t* = 3 min and iii) *t* = 120 min after printing. b) Fluorescence intensity at the filament center and 350 µm outside filament center. c) Change in cross‐section fluorescence intensity over time. d) i) HA filament printed without any cross‐linker in 3% (w/v) alginate at ii) *t* = 3 min and iii) 120 min after printing. e) Fluorescence intensity at the filament center and 350 µm outside the filament center. f) Change in cross‐section fluorescence intensity over time. g) i) HA/PEG‐Az_4_ filament printed in 3% (w/v) alginate at ii) *t* = 3 min and iii) 120 min after printing. h) Intensity at the filament center and 350 µm outside the filament center. i) Change in cross‐section fluorescence intensity over time. j) Effect of printing pressure on filament diameter at constant printing speed of 3 mm s^−1^ and a pressure of i) 10 kPa, ii) 13 kPa, iii) 16 kPa, iv) 19 kPa, v) 22 kPa and vi) 25 kPa. k) Effect of printing speed on filament diameter at constant printing pressure of 10 kPa and speed of i) 3 mm s^−1^, ii) 6 mm s^−1^, iii) 9 mm s^−1^, iv) 12 mm s^−1^, v) 15 mm s^−1^ and vi) 18 mm s^−1^. Scale bars: 250 µm.

In addition to facilitating the printing of filaments, the ATPS strategy prevented dissolution of the filaments during the cross‐linking reaction. Whereas filaments printed in buffer (25 mM HEPES) dissolved completely in 30–60 minutes (**Figure** **2**a–c), filaments printed in 3% (w/v) alginate remained intact for hours even when printed without any cross‐linker (Figure 2d–f). Filaments printed in 3% (w/v) alginate with either of the three different cross‐linkers thus retained their structure and could be incubated in the alginate solution until fully cross‐linked (Figure 2g–i), resulting in mechanically robust and elastic hydrogel filaments that could be extracted from the support bath using, for example, tweezers (Figure 1b). The minor decrease in fluorescence intensity of the ATPS stabilized filaments (Figure 2f,i), is a result of the diffusion of unattached Cy‐dyes and photobleaching during imaging acquisition.

To investigate the possibilities to control the thickness of the printed filaments, printing pressure and speed were varied using a constant needle gauge (25G). Increasing the pressure from 10 to 25 kPa while keeping the printing speed constant at 3 mm/s resulted in an increase in filament diameter from about 475 to 1060 µm (Figure 2j). Increasing the printing speed from 3 to 18 mm/s while keeping the pressure constant at 10 kPa resulted in a decrease in filament diameter from 475 to 160 µm (Figure 2k). The thickness of the filaments could thus, as expected,^[^ ^29^, ^30^ ^]^ be controlled and varied over an extensive range (160 – 1060 µm) with high precision and reproducibility (Figure S3, Supporting information). This size range corresponds to key functional units across multiple organ systems, making it well‐suited for engineering ductal, tubular, or aligned architectures including small arterioles to venules and even small arteries, as well as peripheral nerve bundles, glandular and epithelial ducts, bronchioles, lymphatic vessels, and aligned muscle fibers. Except for the amount of hydrogel and the size of the needle/cartridge and the support bath, we did not notice any technical limitations with respect to the length of the filaments that could be produced.

### 2.2 REFRESH Printing of Alginate/PEG Hydrogels

**Figure 3:**
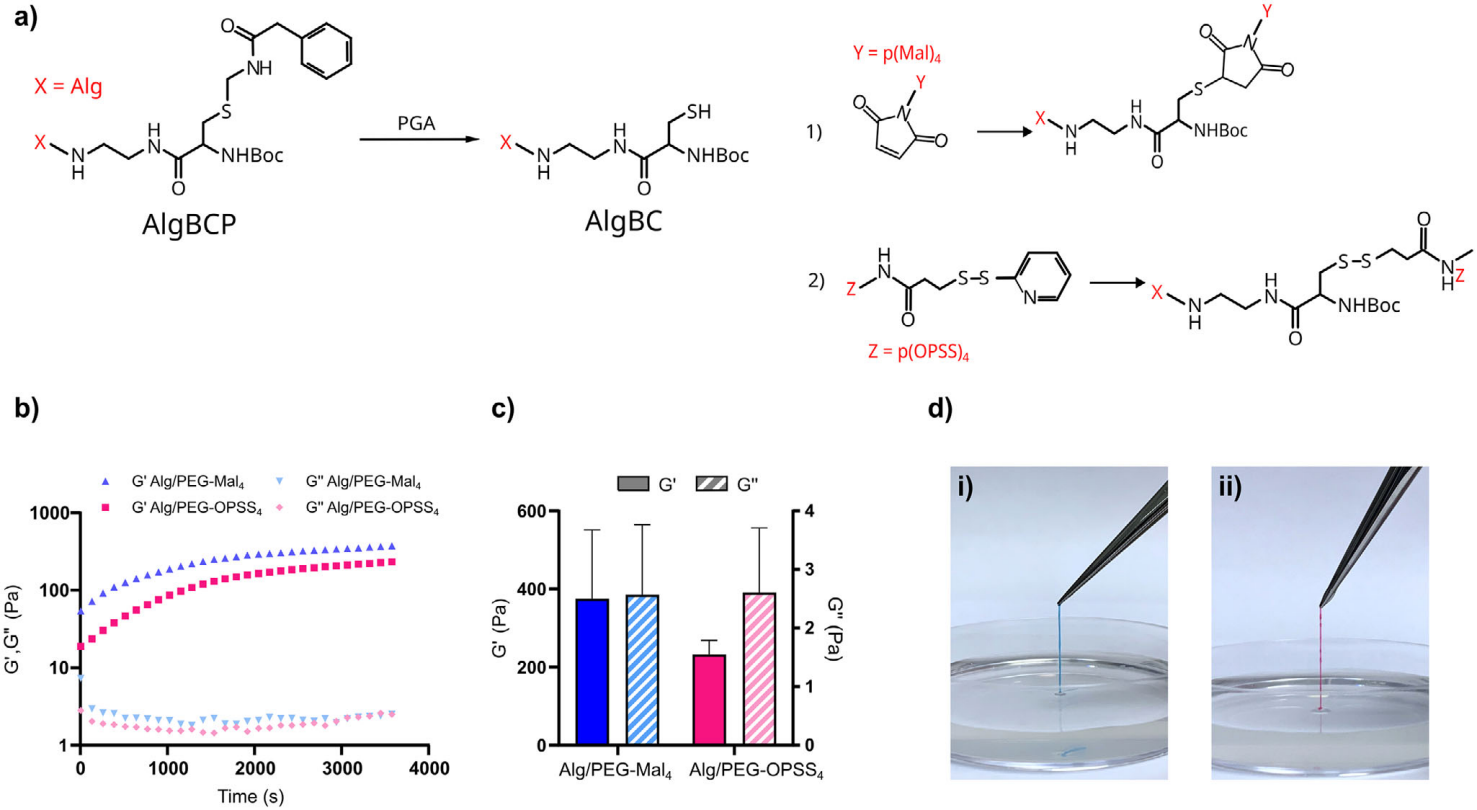
a) Schematic illustration of the deprotection of Alg‐BCP and cross‐linking with either PEG‐Mal_4_ or PEG‐OPSS_4_. b) Cross‐linking kinetics of Alg/PEG hydrogels after the addition of PGA. c) G′ and G″ at the end of gelation. d) Alg‐BCP filaments cross‐linked using i) PEG‐Mal_4_ (blue) or ii) PEG‐OPSS_4_ (red).

To further investigate the versatility of the REFRESH strategy for other bioink materials, we explored the possibility to print alginate/PEG (Alg/PEG) hydrogel filaments. Alginate was functionalized with a Phamc‐protected cysteine (Cys‐Phacm) residue (Alg‐BCP) and cross‐linked using either four‐arm PEG‐maleimide (PEG‐Mal_4_) or four‐arm PEG‐orthopyridyl disulfide (PEG‐OPSS_4_) (**Figure** **3**a). The thiol moiety in Cys‐Phacm is non‐reactive until deprotected by the enzyme penicillin G acylase (PGA).^[^ ^31^, ^32^ ^]^ Alg‐BCP can thus be combined with various thiol‐reactive cross‐linkers without initiating cross‐linking until the addition of PGA. For filament fabrication, Alg‐BCP was carefully mixed with PEG‐Mal_4_ or PEG‐OPSS_4_ at a [Mal]:[SH] or [OPSS]:[SH] stoichiometric ratio of 1:10 prior to printing into the 3% (w/v) alginate REFRESH solution supplemented with 0.1 mg/ml of PGA. Both PEG‐Mal_4_ and PEG‐OPSS_4_ triggered gelation rapidly after the addition of PGA (Figure 3b), generating Alg/PEG hydrogels with similar mechanical properties as the HA/PEG hydrogels (Figure 3c). Albeit the tendency for ATPS is likely lower when printing an Alg/PEG hydrogel precursor into an alginate solution, it was sufficiently pronounced to stabilize the filaments during the rapid PGA‐mediated Cys‐Phacm deprotection and subsequent thiol‐maleimide/thiol‐OPSS cross‐linking process. As a result, defined and mechanically robust Alg/PEG hydrogel filaments were obtained for both the PEG‐Mal_4_ and PEG‐OPSS_4_ cross‐linked hydrogels that could be manually extracted from the alginate solution (Figure 3d).

### 2.3 Mechanical Properties of the HA/PEG Filaments

**Figure 4:**
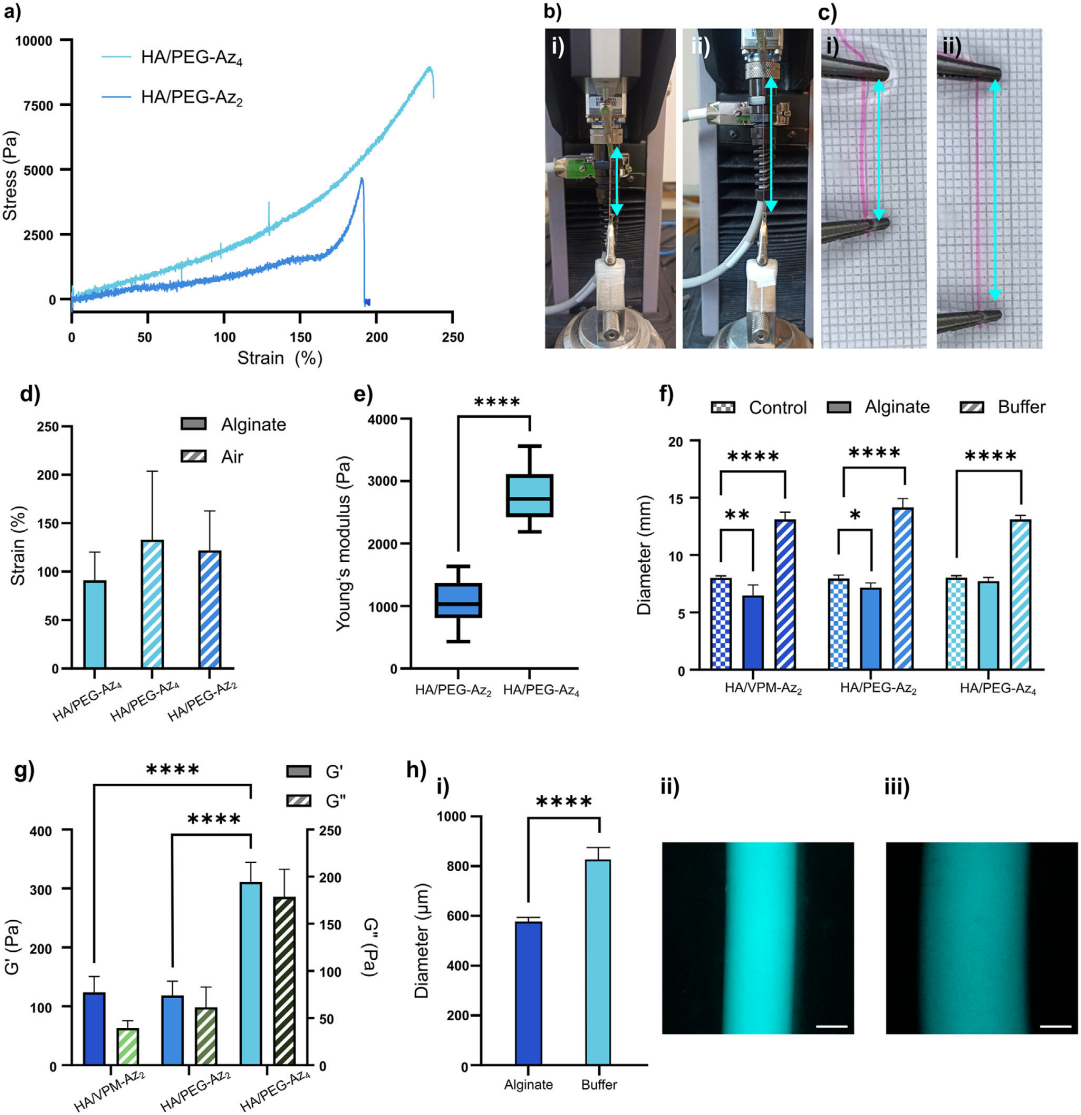
a) Stress/strain curve of HA/PEG filaments. The strain at break of HA/PEG‐Az_4_ and HA/PEG‐Az_2_ hydrogel filaments was 130 ± 60% and 120 ± 40%, respectively. b) Tensile testing of filaments conducted in air: i) start, ii) prior to rupture. c) Manuel tensile testing of filaments in 3% (w/v) alginate: i) Start, ii) prior to rupture. d) Comparison between filament strains when measured in air or alginate. e) Young's modulus of filaments measured in air. f) Swelling/shrinking of hydrogel discs submerged in either 25 mM HEPES buffer or 3% alginate. g) G′ and G″ of hydrogels after submersion in 3% (w/v) alginate. h) Filament diameter expansion when i) moved from 3% (w/v) alginate to buffer. ii) Filament in 3% alginate. iii) Filament in 25 mM HEPES buffer. Scale bars: 250 µm.

Because of the elastic properties of the hydrogels, the filaments were able to withstand significant strain. The strain at break of HA/PEG‐Az_4_ and HA/PEG‐Az_2_ filaments was 130 ± 60% and 120 ± 40%, respectively, when measured using a tensile testing machine in air (**Figure** **4**a,b; Figure S4, Supporting Information). Similar results were obtained when the filaments were subjected to manual tensile testing when submerged in a 3% (w/v) alginate solution (Figure 4c), with a yield strain of 91 ± 23%. Water evaporation during tensile testing did not have a major impact on the mechanical properties of the hydrogels but could impact the overall reproducibility, likely contributing to the relatively high standard deviations when measured in air (Figure 4d). The measured Young's modulus of the filaments was 2.8 ± 0.4 kPa for HA/PEG‐Az_4_ hydrogels filaments and 1.1 ± 0.3 kPa for HA/PEG‐Az_2_ hydrogel filaments (Figure 4e), which is within the expected range based on the rheological data obtained from the bulk hydrogels. The higher modulus seen for HA/PEG‐Az_4_ is a result of the higher cross‐linking density of the hydrogels, compared to HA/PEG‐Az_2_ hydrogels formed with the linear cross‐linker.

The mechanical properties of hydrogels depend on the hydration level, which in turn is influenced by the osmotic pressure. To explore the effect of the presence of alginate on the swelling of the hydrogels, fully cross‐linked hydrogel discs were submerged in either buffer (25 mM HEPES) or in 3% (w/v) alginate for 2 hours followed by rheological characterization. Hydrogel discs submerged in buffer showed an influx of water due to the osmotic pressure difference, resulting in swelling, whilst hydrogel discs submerged in 3% (w/v) alginate showed an outflux of water and shrinking, resulting in a decrease in stiffness (Figure 4f; Figure S5, Supporting Information). The hydrogels submerged in alginate were 30–50% less stiff than the corresponding hydrogels swollen in buffer alone with a G′ of 0.12 ± 0.03, 0.12 ± 0.02, and 0.3 ± 0.03 kPa for the HA/VPM‐Az_2_, HA/PEG‐Az_2_, and HA/PEG‐Az_4_, respectively (Figure 4g; Figure S2, Supporting Information). Consequently, transferring filaments to buffer or cell culture medium caused them to swell (Figure 4h), resulting in a slight loss in flexibility. However, this process was reversible, and filaments regained their full flexibility when returned to the alginate solution. Irrespective of whether submerged in buffer, cell culture media, or in an alginate solution, the filaments were sufficiently robust and elastic to allow for manual handling after cross‐linking (Movie S1, Supporting Information).

### 2.4 Shape Memory Properties of HA/PEG Filaments

**Figure 5:**
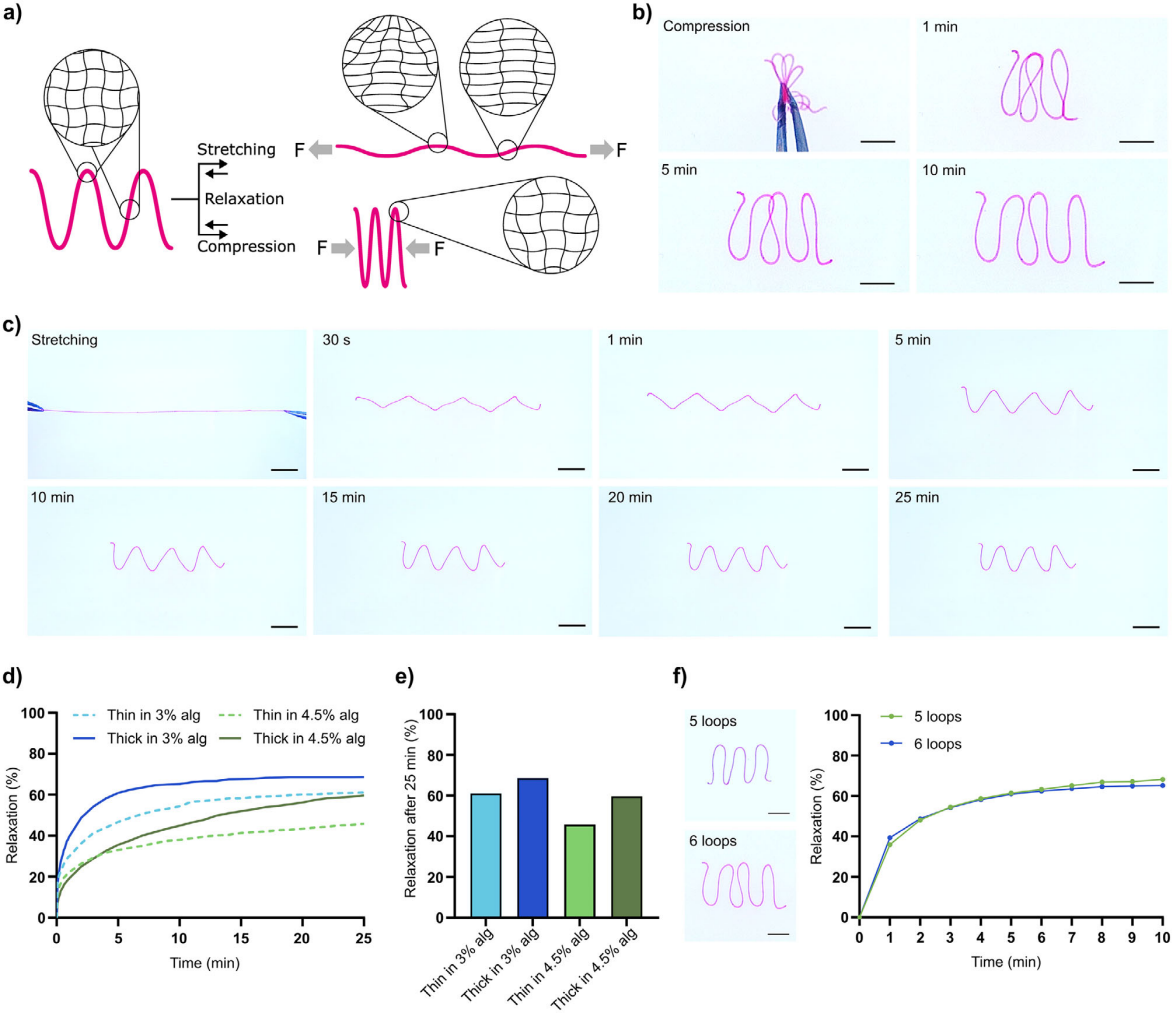
a) Schematic illustration of the shape memory properties of HA/PEG‐Az_4_ filaments when subjected to stretching and compression and the resulting anisotropic deformation of the polymer network that result in shape recovery. b) Compression‐induced deformation and relaxation over 10 minutes. c) Stretching and subsequent relaxation over 25 minutes. d) Quantitative analysis of relaxation kinetics for thin and thick filaments in 3% or 4.5% alginate slurry over time. e) Shape recovery after 25 min relaxation. f) Effect of loop number on relaxation kinetics. Filaments printed with five and six loops were compared over 10 minutes. Scale bars b,f) 5 mm. Scale bars c) 10 mm.

Interestingly, we observed that filaments that were mechanically deformed with respect to the printed shape tended to relax back to the original shape over time, indicating a distinct shape memory behavior (Movie S2, Supporting Information). The shape memory properties of the filaments likely stem from the homogenous and efficient cross‐linking of the HA backbone and the elasticity of the materials, that is independent of the printed geometry. Any deformation of the printed shapes results in an anisotropic deformation of the polymer network and buildup of stress that increases with the applied force and the curvature of the geometry of the filaments as schematically illustrated in **Figure** **5**a.^[^ ^33^ ^]^ Moreover, when filaments are subjected to compression, the osmotic repulsion due to molecular crowding effects likely contributes to the relaxation and shape recovery.^[^ ^34^ ^]^

To investigate the shape memory of the HA/PEG‐Az_4_ filaments, filaments were printed in a zigzag geometry and subjected to manual stretching or compression. All filaments relaxed close to the initial shape when the deforming forces were released (Figure 5b,c). Compression‐induced deformation enabled progressive relaxation over 10 minutes (Figure 5b). The filaments transitioned from a compact conformation to an extended shape, with close to full recovery back to the original printed geometry. Similarly, after stretching the filaments, they gradually relaxed into their original shape over a time period of about 15–25 min (Figure 5c). Quantitative analysis of the relaxation kinetics (Figure 5d) revealed that filaments that were stretched in a 3% alginate solution exhibited higher degree and faster relaxation compared filaments stretched in a 4.5% alginate solution, likely due to the higher viscosity of the solution and potentially due to the outflux of water from the filaments due to the difference in polymer density between the filaments and the alginate solution resulting in loss of filament elasticity. Thicker filaments (ø ≈640 µm) demonstrated faster and a higher degree of relaxation compared to thinner filaments (ø ≈550 µm). After 25 minutes, the maximum shape recovery was 61% and 69% for the thin and thick filament in 3% alginate solution, respectively, whereas the recovery was 46% and 60% for the thin and the thick filament in the 4.5% alginate solution (Figure 5e). These results suggest that both the viscosity of the surrounding matrix and the filament thickness influence the rate and extent of shape memory mediated relaxtion. Thicker filaments likely possess greater stored elastic energy, facilitating a higher degree of relaxation. Additionally, the influence of loop numbers on relaxation was evaluated (Figure 5f). Filaments with five and six loops showed close to identical relaxation profiles over 10 minutes, with no significant difference in relaxation kinetics between the two configurations. This suggests that shape memory is governed by the material properties and the properties of the surrounding matrix, but not the initial loop number. Complete (100%) relaxation was never observed, likely due to network rearrangements and stress‐induced breaking of some polymer chains and/or cross‐links. This was confirmed by exploring the effect of repeated stretching of the filaments. After a second cycle of stretching, a reduction in relaxation can be seen for both thin and thick filaments (Figure S6, Supporting Information). The loss of memory function can thus be prevented by operating the applied stress below the yield point of the materials, and likely by optimizing the printed geometries, which can enable development of novel hydrogel‐based mechanical actuators.

### 2.5 Postprocessing and Bioprinting with Embedded Cells

**Figure 6:**
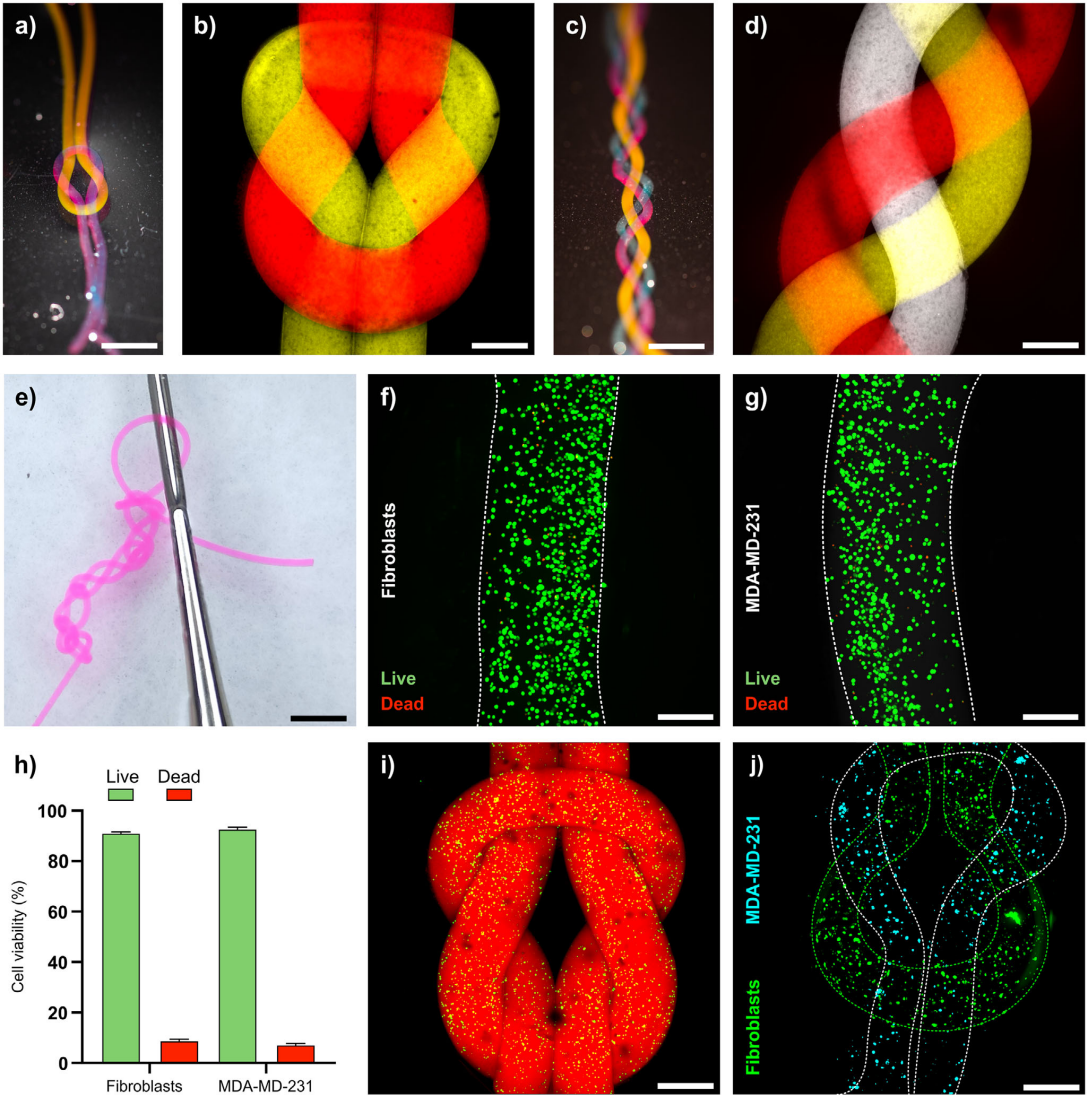
a) Photograph and b) spinning disc confocal microscopy image of a HA/PEG filament knot, stained with Cy5‐Az/Cy3‐Az. c) Photograph and d) spinning disc confocal microscopy image of a HA/PEG filament braid, stained with Cy7‐Az/Cy5‐Az/Cy3‐Az. e) HA/PEG filament knitting (crocheting), stained with Cy3‐Az. Live (green) and dead (red) staining of f) human primary fibroblasts and g) MDA‐MD‐231 breast cancer cells cultured in HA/PEG‐Az_4_ filaments. h) Cell viability after 24 hours in culture. i) Spinning disc confocal microscopy image of a HA/PEG filament knot stained with Cy5‐Az with embedded human primary fibroblast (green, phalloidin). j) Multicellular hydrogel filament structure comprising one filament with human primary fibroblast (green) and a second hydrogel filament with MDA‐MD‐231 cells (cyan). Scale bars a,c,e) 5 mm. Scale bars b,d,f,g,i,j) 250 µm.

The possibility to manually process and reroute the hydrogel filaments was demonstrated by knotting and braiding (**Figure** **6**a–d). The filaments were even robust enough to sustain crocheting (Figure 6e), thus showing the potential for creating complex structures that cannot be manufactured by conventional extrusion‐based 3D bioprinting (Figure S7, Supporting Information). We have previously demonstrated that the HA/PEG and HA/VPM hydrogels can support proliferation of a wide range of different cells.^[^ ^24^, ^27^ ^]^ To explore the cell compatibility of the filament and the manual processing of the filaments, the hydrogels were first functionalized with cRGD‐Az to promote cell‐hydrogel interactions.^[^ ^26^, ^35^, ^36^ ^]^ Primary human fibroblast and MDA‐MB‐231 breast cancer cells were then combined with the hydrogel components and bioprinted into filaments in 3% (w/v) alginate solution supplemented with Hank's Balanced Salt Solution (HBSS). After cross‐linking for 2 hours, the filaments were transferred to cell culture media supplemented with FBS and 3% (w/v) alginate to maintain the mechanical flexibility of the filaments. A live/dead assay was conducted after 24 hours to assess the cell viability of cells in the printed filaments, showing 91 ± 0.5% viability for fibroblast and 93 ± 0.6% viability for MDA‐MA‐231 cells (Figure 6f–h), demonstrating excellent cell viability after bioprinting.

The viscoelastic properties of the cell‐laden hydrogels were not significantly different to hydrogels without cells, and the cell‐laden filaments could thus be rerouted and processed manually just as the non‐cell containing filaments (Figure 6i,j; Figure S8, Supporting Information). Cells cultured in the knotted filaments for up to 7 days showed high cell viabilities (>90%) for both fibroblast and MDA‐MB‐231 cells throughout the culture period (Figure S8, Supporting Information). The possibilities to join two filaments with identical or different cell types by knotting or using other textile engineering technologies could facilitate fabrication of unique multicellular constructs and advanced co‐cultures.,

### 2.6 Protease Degradation of Filaments

**Figure 7:**
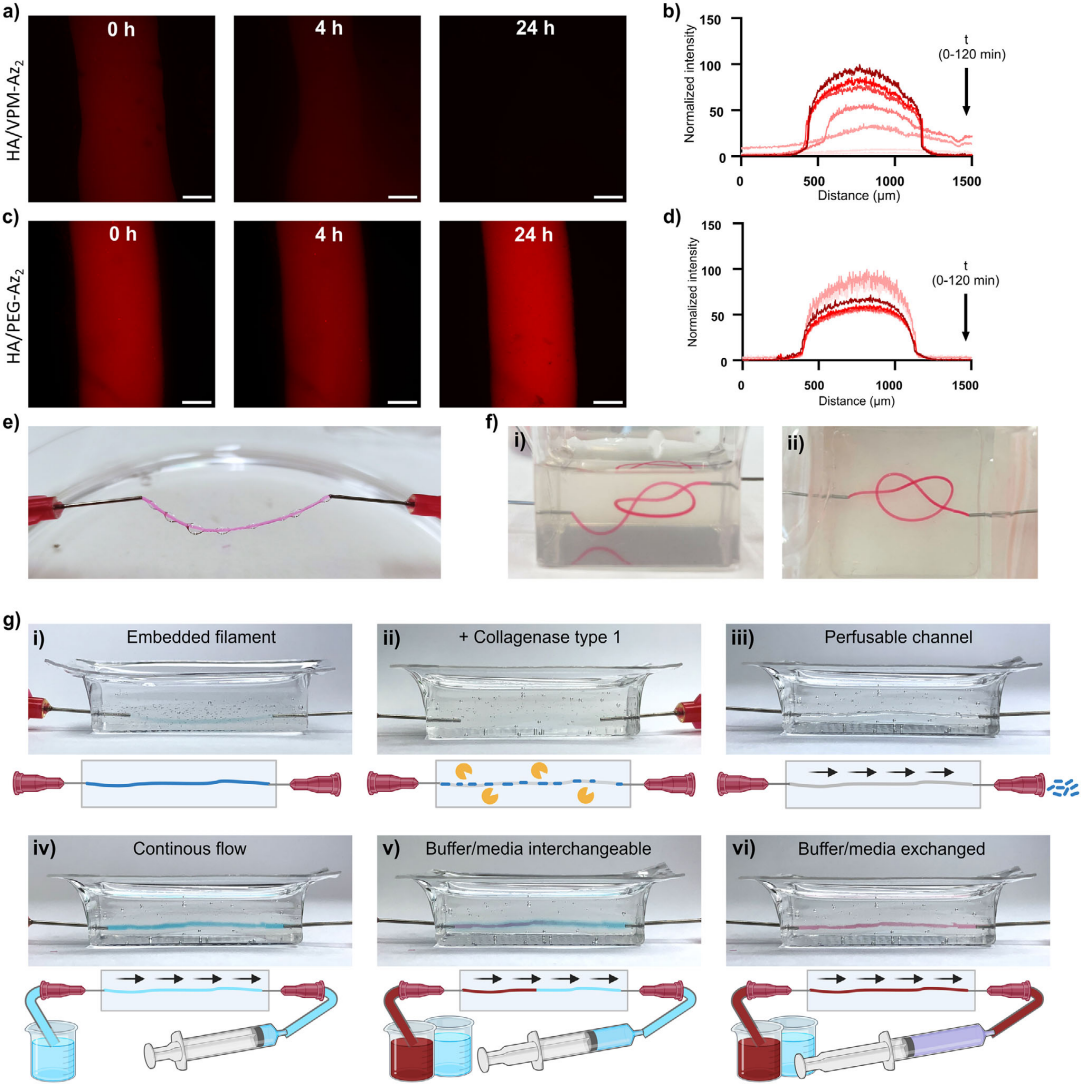
a) Degradation of HA/VPM‐Az_2_ filaments after addition of 0.5 mg mL^−1^ collagenase type 1: i) 0 h, ii) 4 h, iii) 24 h. b) Change in cross‐section fluorescence intensity over time. c) HA/PEG‐Az_2_ filaments after addition of 0.5 mg mL^−1^ collagenase type 1: i) 0 h, ii) 4 h, iii) 24 h. d) Change in cross‐section fluorescence intensity over time. e) HA/VPM‐Az_2_ filament connected to syringes. f) HA/VPM‐Az_2_ filament knot embedded in an alginate hydrogel. i) side view, ii) top view. g) Collagenase type 1 degradation a HA/VPM‐Az_2_ filament embedded in an alginate hydrogel: i) prior addition of collagenase type 1; ii) after collagenase type 1 degradation of HA/VPM‐Az_2_ filament; iii) removal of degradation products leaving a hollow channel; iv‐vii) perfusion of the channel (flow from left to right) using a peristaltic pump. h) HA/VPM‐Az_2_ filament embedded in alginate. i) prior collagenase type 1, ii) after removal degradation products, iii) perfusion. Scale bars: 250 µm.

The HA/VPM hydrogels are responsive to a wide range of proteases, including matrix metalloproteinases.^[^ ^24^ ^]^ In addition to degradation by endogenous proteases secreted by encapsulated cells, we have previously demonstrated that addition of collagenase type 1 results in complete and rapid degradation of the hydrogels.^[^ ^24^ ^]^ Consequently, HA/VPM‐Az_2_ filaments were rapidly degraded upon the addition of 0.5 mg mL^−1^ collagenase type 1, whereas the HA/PEG‐Az_2_ filaments remained intact (**Figure** **7**a–d). The possibility to degrade the filaments proteolytically makes them attractive as a sacrificial structure for creating perfusable channels in protease‐resistant hydrogels, such as alginate. HA/VPM‐Az_2_ filaments were thus attached in both ends to syringes (Figure 7e) and embedded in a 2% alginate hydrogel cross‐linked with CaSO_4_ (Figure 7f, Movie S3, Supporting Information). Addition of 0.5 mg/ml collagenase type 1 resulted in rapid dissolution of the HA/VPM‐Az_2_ hydrogel leaving a perfusable tubular channel (Figure 7f,g, Movies S4, S5, Supporting Information). The possibility to manually arrange the filaments in the alginate hydrogel prior to proteolytic degradation enabled fabrication of perfusable tubular architectures with complex 3D geometries (e.g., knots) that would be challenging to fabricate using conventional extrusion‐based 3D bioprinting techniques (Figure 7f; Figure S9, Supporting Information). The degradation and removal of sacrificial filaments by either cell‐friendly exogenous proteases or by proteases secreted by the cells embedded in either the filaments or in the alginate hydrogel can facilitate in situ cell‐mediated generation of vasculature‐like constructs. Moreover, the size of the channels can be tuned by using filaments with different diameters to tailor dimensions of the perfusable structures (Figure 2j,k).

## 3 Conclusion

We present an ECM mimicking hydrogel system and an extrusion‐based 3D bioprinting technology (REFRESH) that enable fabrication of highly elastic and mechanically robust suspended cell‐laden hydrogel filaments. The hydrogels were cross‐linked by SPAAC using either PEG‐ or a peptide‐based protease‐cleavable cross‐linker and were printed in an alginate supported bath. The dimensions of the filaments could be tailored with high precision by tuning of the printing parameters. The printed filaments were efficiently stabilized by ATPS during and after printing to maintain filament integrity during the cross‐linking process. In stark contrast to most ECM mimicking hydrogels optimized for 3D cell culture, which are typically very soft and fragile, the resulting hydrogel filaments were mechanically robust and highly elastic with a strain of break exceeding 100%. The unique mechanical properties of the filaments enabled manual handling and postprocessing using textile‐inspired engineering techniques into intricate 3D architectures, by for example knotting, braiding, and crocheting. Such complex intertwined 3D structures are currently not possible to fabricate using conventional extrusion bioprinting techniques. Moreover, the bioorthogonal cross‐linking chemistry used here allowed for encapsulation and culture of both primary human cells (dermal fibroblasts) and breast cancer cells (MDA‐MB‐231) in the filaments, with high cell viabilities. The cell‐laden filaments did not lose their mechanical flexibility, which was demonstrated by joining different filaments with different cell types by knotting. Although carried out manually in the present work, textile inspired techniques can be automatized and are scalable, which could enable fabrication of large hydrogel‐based cell multicellular constructs for a wide range of biomedical applications. Because of the elastic properties of the hydrogels and the homogenous cross‐linking, the printed filaments displayed distinct shape memory properties. Relaxation after mechanical deformation of filaments printed in a specific geometry resulted in gradual shape recovery, irrespectively whether the filaments were subjected to compression or stretching. When exchanging the PEG‐based cross‐linkers for a peptide‐based protease‐cleavable cross‐linker, protease‐degradable filaments were obtained. Because of the mechanical robustness of the protease degradable filaments, they could be embedded in protease inert hydrogels in elaborate geometries and used us sacrificial templates for generating perfusable tubular structures. Manual rerouting of filaments prior degradation with cytocompatible exogenous proteases or endogenous cell‐secreted proteases can provide new means to facilitate the fabrication of complex vascular‐like architectures. In summary, the hydrogels and the REFRESH technique presented here significantly broadens the potential of extrusion‐based bioprinting, offering a powerful tool for generating sophisticated and complex 3D cell‐laden structures.

## 4 Experimental Section

### Materials

If not stated otherwise, all chemicals were obtained from Merck Life Science AB (Stockholm, Sweden) and used without further modification.

### HA‐BCN Synthesis

Sodium hyaluronate (HA), 500 mg, 100–150 kDa (Lifecore Biomedical Inc., Chaska, USA) was dissolved in 40 mL 100 mM MES buffer, pH 7.0. In a separate container, 5 mL of acetonitrile and 1 mL of MilliQ water (MQ) (18.2 MΩ cm^−1^) were mixed, and 100 mg of N‐[(1R,8S,9s)‐Bicyclo[6.1.0]non‐4‐yn‐9‐ylmethyloxycarbonyl]‐1,8‐diamino‐3,6‐dioxaoctane (BCN‐NH_2_), 83 mg of 1‐Hydroxybenzotriazole hydrate (HoBt), and 236 mg of N‐(3‐Dimethylaminopropyl)‐N′‐ethylcarbodiimide hydrochloride (EDC) were added. When dissolved, the two solutions were combined, and the pH was adjusted to 7.0. The solution was then left to react on an orbital shaker at room temperature for 24 hours and then transferred to a dialysis bag, 6–8 kDa (Spectra/Por RC, Spectrum Laboratories, New Brunswick, USA) and dialyzed against 10% acetonitrile (ACN) in MQ for 24 hours, with the dialysis solution being changed twice and the dialyzed against MQ for 7 days, with the solution being changed twice a day. Lastly, the purified product was diluted 1:1 with MQ, and the pH was adjusted to 6.5 before freeze‐drying.

### VPM and cRGD Peptide Synthesis

cRGD, with the sequence c(RGDfK(Az)), was synthesized as described previously.^[^ ^37^ ^]^ VPM‐Az_2_, with the sequence Ac‐(K(Az)‐GRDVPMSMRGGDR‐K(Az))‐CONH_2_, was synthesized using a microwave‐supported automated peptide synthesizer (Liberty Blue, CEM, Matthews, North Carolina, USA) using conventional fluorenylmethyloxycarbonyl (Fmoc) chemistry and was performed on a 250‐micromole scale using a Rink amide resin (ProTide LL, CEM) with a loading capacity of 0.19 mmol g^−1^. The amino acids were provided by Iris Biotech GmbH in Marktredwitz, Germany, and was coupled using a five‐times excess of the amino acid, N,N′‐Diisopropylcarbodiimide (DIC) as the coupling agent, and a ten‐times excess of Oxyma Pure as the base and microwave heating at 90 °C for a duration of two minutes. The coupling steps were repeated twice. The Fmoc group was subsequently removed using a 20% solution of piperidine in DMF (dimethylformamide) at 90 °C for 60 seconds. After the final removal of the Fmoc group, N‐terminal acetylation was performed by exposing the peptide to a 50% solution of acetic anhydride in DMF for 60 minutes. The unrefined peptide was isolated via cleavage and complete deprotection using a solvent mixture of trifluoroacetic acid (TFA), water, and triisopropylsilane (TIS) in a 95:2.5:2.5 (v/v/v) ratio for three hours. It was subsequently filtered, concentrated, and subjected to two rounds of precipitation in ice‐cold diethyl ether. Purification was carried out using a reversed‐phase C‐18 column (ReproSil Gold), connected to a Dionex Ultimate 3000 LC HPLC system (Thermo Fisher Scientific, Waltham, Massachusetts, USA). After concentration and lyophilization, the peptide's purity was verified using reversed‐phase HPLC and MALDI‐ToF MS using a Bruker UltrafleXtreme (Bruker Daltonics, Billerica, Massachusetts, USA).

### Hydrogel Synthesis and REFRESH Printing of HA/PEG and HA/VPM

For all experiments, 20 mg mL^−1^ of HA‐BCN was dissolved while stirring in sterile PBS supplemented 1% antibiotic‐antimycotic (10000 units mL^−1^ of penicillin, 10,000 µg mL^−1^ of streptomycin and 25 µg mL^−1^ amphotericin B).To the dissolved HA‐BCN solution (20 mg mL^−1^), 10 mg mL^−1^ Xanthan gum (XG) was added together with either 50 µM Cy3‐Azide (Cy3‐Az), Cy5‐Azide (Cy5‐Az), or Cy7‐Azide (Cy7‐Az) (Lumiprobe GmbH, Hannover, Germany) and left to dissolve and react for 24 hours on a magnetic stirrer. To form a hydrogel, the polymer solution (HA‐BCN 20 mg mL^−1^ + XG 10 mg mL^−1^) was combined with a cross‐linker‐solution at a 7:1 ratio. To create either a protease‐degradable or a non‐protease degradable hydrogel, different cross‐linkers were selected. For a protease degradable hydrogel 20 mg mL^−1^ VPM‐peptide (VPM‐Az_2_), and for a non‐protease degradable hydrogel, either 12.34 mg mL^−1^ PEG‐Az_2_ (Creative PEGworks, Chapel Hill, North Carolina, USA) or 56 mg mL^−1^ PEG‐Az_4_ (Creative PEGworks, Chapel Hill, North Carolina, USA) have been used. All cross‐linkers were dissolved in sterile PBS supplemented with 1% antibiotic‐antimycotic. The polymer and cross‐linker solutions were mixed thoroughly and transferred to a 25G printing needle. Filaments were printed in either 25 mM HEPES pH 7.4 buffer or a 1–5% alginate solution dissolved in filtered 25 mM HEPES pH 7.4 using a BioX 3D bioprinter (Cellink AB, Gothenburg, Sweden). The printed filaments were incubated for a minimum of 2 hours at 37 °C to complete the filament cross‐linking prior to subsequent processing.

### Synthesis of Alg‐BCP

AlgBCP was synthesized as previously described.^[^ ^31^ ^]^ Briefly, Boc‐l‐Cys(Phacm)‐OH (BCP, 13.6 mmol) was dissolved in chloroform and reacted with ethyl carbodiimide hydrochloride (EDC, 15.0 mmol), N‐hydroxy succinimide (NHS, 15.0 mmol), and diisopropylethylamine (30 mmol) to form a cysteine succinimide ester. Ethylenediamine (1.4 mol) was then added and stirred overnight. The product was washed, dried, filtered, concentrated, and purified using C18 column chromatography with an acetonitrile gradient. For conjugation to alginate, 250 mg of alginate was dissolved in MES buffer and mixed with acetonitrile. BCP (0.5 mmol), HOBt (1 mmol), and EDC (2 mmol) were dissolved in MES buffer/acetonitrile, and the solutions were combined. The reaction proceeded overnight at pH 7, followed by 72 hours of dialysis against acetonitrile and water.

### REFRESH Printing of Alg‐BCP/PEG Filaments

The Alg/PEG‐Mal_4_ ink was prepared containing 1% AlgBCP, 0.8% 4‐arm PEG‐maleimide (10 kDa, Creative PEGWorks) and 328 µM Cyanine5‐maleimide (Lumiprobe) in HEPES (50 mM, pH 7.4) and Alg/PEG‐OPSS_4_ ink contained 1% AlgBCP, 0.8% 4‐arm PEG‐orthopyridyl disulfide (OPSS) (10 kDa, Creative PEGWorks) and 400 µM Cyanine3‐maleimide (Lumiprobe) in HEPES (50 mM, pH 7.4). The support bath consisted of 3% (w/v) alginate supplemented with 0.1 mg mL^−1^ of PGA (10 mg mL^−1^, Biosynth). The printing was done at 5 kPa pressure, 5 mm s^−1^ printing speed and 100 ms pre‐flow.

### Fluorescence Correlation Spectroscopy

Fluorescence correlation spectroscopy^[^ ^38^, ^39^ ^]^ was used to measure the diffusion of Cy5‐Az‐labelled HA‐BCN molecules in the presence of varying concentrations of alginate. The measurements were performed on a Zeiss LSM780 confocal microscope equipped with a 63x water immersion lens with numerical aperture 1.0, using the 633 nm He‐Ne laser line and acquisition software supplied by the microscope manufacturer. In brief, a Petri dish was placed on the microscope stage and 3 – 5 mL of solution added until the tip of the objective lens was submerged. At each alginate concentration, fluorescence fluctuations were recorded at 2% laser power for a total of 100 s, using 10 runs of 10 s. Measurements at each alginate concentration were repeated on three different samples. Fluorescence fluctuations were inspected offline and records with unstable count rate excluded from further analysis. The remaining records were averaged and theoretical models for 3D diffusion fitted to the data using Matlab's curve fitting toolbox (The Mathworks, R2024a). Adequate fitting required models for anomalous diffusion^[^ ^40^ ^]^ as expected from the crowded molecular environment in the present experiments. In addition, most records showed multiple species of fluorescent molecules, likely representing low concentrations of fast‐diffusing free Cy5 molecules as well as the more slowly diffusing Cy‐5‐HA‐BCN molecules. Data in Figure 1f show the diffusion time of the slowly diffusing component only. Shorter diffusion times mean that molecules move faster through the confocal microscope's detection volume.

### Rheology

A 0.5 mm Polyethylene terephthalate glycol (PET‐G) sheet with circular molds measuring 8 mm in diameter was pressed against a glass slide covered with parafilm. In each mold, 60 µL of hydrogel was added to form hydrogel discs. The mold was placed in a petri dish with damp tissue paper and sealed with parafilm before being incubated for 2 hours at 37 °C. After 2 hours, the discs were cut out from the molds using a scalpel and hydrated in either 3% alginate dissolved in 25 mM HEPES or 25 mM HEPES buffer only for 24 hours. Rheological measurements were performed using a Discovery Hybrid Rheometer (TA Instruments, New Castle, Delaware, USA) with an 8 mm parallel plate geometry at 25 °C. For all gels, an amplitude sweep with 0.1–1% strain at 1 Hz, a frequency sweep with 1% strain at 0.1–10 Hz, and a time sweep with 1% strain at 1 Hz was performed. The gap for the hydrated gels ranged from 0.4–0.6 mm for the 3% alginate hydrated gels and 0.8–1 mm for the buffer hydrated gels. Gelation time was determined by measuring 45 µL hydrogel sample at 1% strain at 1 Hz for 2 hours at 37 °C using a 20 mm 1° cone geometry. Viscosity was determined by measuring 45 µL solution at a linear shear rate sweep between 0.1–100.

### Tensile Testing

Hydrogel filaments, approximately 60 mm long, 540–650 µm in diameter, hydrated in 3% alginate, were clamped in an MTS Criterion Model 41 electromechanical universal test system (MTS Systems Corporation, Eden Prairie, MN, USA) with a 1 N load cell, by using metal clamps. The initial distance was set to 20 mm. Extension was done at 0.1 mm s^−1^ in air until the filament ruptured. For tensile testing in 3% alginate, the filaments were clamped with tweezers and manually extended until rupture.

### Shape Memory Function

HA/PEG‐Az_4_ filaments were printed at 12–14 kPa in 3% and 4.5% (w/v) alginate. Printed filaments were subjected to manual compression and stretching and the shape recovery was recorded with a camera. Screenshots of the video were taken at defined intervals. Relaxation was calculated using the following expression:

(1) $$\mathrm{Relaxation}\left(\%\right)=100-\frac{\text{Length at timepoint X}}{\text{Length when stretched/compressed}}\times 100$$

### Enzymatic Degradation of Filaments

HA/PEG‐Az_2_ or HA/VPM‐Az_2_ filaments were placed in an Ibidi plate. Additional HA/PEG‐Az_4_ hydrogel was added to the ends of the filaments to secure them to the bottom of the plate. MQ was added to the edges of the Ibidi plate and incubated for 2 hours. 1.5 mL of 2% alginate mixed with 20 mM CaSO_4_ was added to cover the filaments and left to cross‐link for 5 minutes. 1 mL of 25 mM HEPES containing 5 mM CaCl_2_ and 0.5 mg mL^−1^ collagenase type 1 was added on top of the cross‐linked alginate hydrogel. Degradation was measured with a 24‐hour time‐lapse, capturing a z‐stack of approximately 250 slices every 30 minutes, using a Nikon spinning disc microscope.

### Culture of Cells in Filaments

Primary human dermal fibroblasts were obtained from skin biopsies from healthy patients as described in detail elsewhere.^[^ ^24^ ^]^ MDA‐MA‐231 cells were a kind gift from Prof. Charlotta Dabrosin (Division of Surgery, Orthopedics and Oncology (KOO), Linköping University). All experiments involving human tissue and cells were performed under ethical approval from the Swedish Ethical Review Authority (no. 2018/97‐31) and in accordance with ethical standards postulated by Linköping University and Swedish and European regulations. The fibroblast and MDA‐MB‐231 cells were seeded into 75 cm^2^ culture flask and cultured in high‐glucose Dulbecco's modified Eagle's medium with phenol red (DMEM, Gibco Thermo Fisher Scientific, Paisley, UK) or in low‐glucose DMEM without phenol red respectively, each supplemented with 10% fetal bovine serum (FBS) and 1% penicillin‐streptomycin (6000 µg mL^−1^ penicillin, 10 000 µg mL^−1^ streptomycin) (Biowest, Rue de la Caille, France). The media was changed every 2–3 days, and cells were passaged at 80% confluency. The fibroblast and MDA‐MB‐231 cells in this study were used from passage 5–8. The cells were detached using TrypLE Express (1X) for 3 min, followed by 10% FBS containing media to neutralize the enzyme. Cells used for live/dead assay were counted, centrifuged at 350 x G for 5 min and resuspended in media. Cells to be stained with CellTrace (Invitrogen, Thermo Fisher Scientific Waltham, Massachusetts, USA) were prepared according to protocol. Briefly, after being counted, cells were resuspended in warm PBS for a final cell density of 10^6^ cells mL^−1^. 5 mM CellTrace dissolved in DMSO, was added for a final working concentration of 5 µM. Cells and staining solution were incubated for 20 min at 37 °C in the dark. Fibroblasts were stained with CellTrace CFSE and MDA‐MB‐231 were stained with CellTrace Yellow. After incubation, 5 times the initial staining solution of media with 10% FBS was added and again incubated at 37 °C for 5 min. The stained cells were centrifuged and resuspended in 1 mL media. For each print, cell media was aliquoted to a final cell density of 2 million cells mL^−1^. The aliquoted cells were again centrifuged at 350 x G for 5 min, the supernatant was removed, and cells were resuspended in cross‐linker and cRGD. 100 µM cRGD were incorporated into every filament. The cell/cross‐linker/cRGD mixture was then mixed with HA‐BCN + XG and printed as previously described in alginate dissolved in 25 mM HEPES pH 7.4 supplemented with 1x HBSS. The printed filaments and its surrounding solution were incubated at 37 °C, 5% CO_2_, and 95% humidity. After 2 hours of incubation and cross‐linking, the filaments were transferred to 3% alginate in high‐glucose DMEM without phenol red supplemented with 10% FBS and 1% antibiotic‐antimycotic.

### Cell Viability in Printed and Rerouted filaments

The acuate viability of cells in the filaments after printing and knotting was assessed by live/dead staining (Biotium, Fremont, California, USA). Briefly, PEG‐Az_4_ cross‐linked filaments with unstained cells were printed as described above. After incubation in 3% alginate in high‐glucose DMEM without phenol red media supplemented with 1% antibiotic‐antimycotic, for 24 hours, the filaments were transferred to individual wells in a well plate. 800 µL of live/dead staining solution (2 µM calcein AM and 4 µM ethidium homodimer‐1 in PBS) was added to the filaments and incubated at room temperature for 40 minutes in the dark. After incubation, the filaments were transferred back to the alginate/media solution. To quantify the of the number of living cells, a z‐stack of approximately 250 slices with 2 µm increments was acquired using a Nikon spinning disk module. Images where processed, and live/dead cells were counted using Fiji (ImageJ, NIH, US).^[^ ^41^ ^]^ For assessment of long‐term cell viability, cells were expanded to the required confluency, harvested, and resuspended in PEG‐Az₄ and cRGD at a final concentration of 4 × 10⁶ cells mL^−1^ and 400 µM cRGD. Cell‐laden HA/PEG‐Az₄ filaments were printed as described above at 14 kPa pressure, 3 mm s^−1^ printing speed and 20 ms preflow. Following printing, constructs were cross‐linked for 1.5 hours at 37 °C, 5% CO₂. Post‐cross‐linking, filaments were manually knotted into two configurations: (i) MDA–fibroblast and (ii) MDA–MDA. Knotted constructs were transferred to 12‐well plates, and filament ends were secured using drops of HA/PEG‐Az₄ hydrogel. Knotted constructs were maintained under standard culture conditions for 1, 4, or 7 days, with media changes every 2–3 days. At designated time points, constructs were washed with PBS and stained with Zombie Red (1:1000) (Biolegend, San Diego, USA) and Hoechst 33 342 (1:10000) (Invitrogen, California, USA) in PBS for 30 minutes at room temperature, followed by two PBS washes. Samples were then fixed with 4% formaldehyde for 30 minutes, washed three times with PBS, and stored until imaging. Samples were imaged using a Stellaris 5 confocal microscope. Fluorescence channels were set as follows: Alexa Fluor 594 for Zombie Red, Hoechst 33 342 for nuclear staining. Images were acquired as tile scans with z‐stacks. Image analysis was performed using ImageJ (version 2.16.0/1.54 g). Z‐stacks were projected to maximum intensity, and brightness was adjusted to a minimum of 0 and a maximum of 80 for Hoechst, and a minimum of 50 and a maximum of 150 for Zombie Red. Thresholds were applied as follows: Hoechst (min: 20, max: 115) and Zombie Red (min: 90, max: 190). Particle analysis was conducted with size and circularity parameters set to 7.5–35 µm (circularity: 0.5–1) for Hoechst and 20–70 µm (circularity: 0.5–1) for Zombie Red.

### Generation of Perfusable Tubular Structures

Straight hollow channels were generated by preparing filaments cross‐linked by VPM‐Az_2_. The filaments were attached to needles and submerged in a 2% (w/v) alginate slurry mixed with 20 mM CaSO₄. The alginate hydrogel was allowed to cross‐link for 5 minutes at room temperature. To degrade the embedded channels, 1 mL of a 0.5 mg mL^−1^ collagenase type 1 solution in 25 mM HEPES buffer supplemented with 5 mM CaCl₂ was added on top of the alginate and incubated at 37 °C for 24 hours. To create hollow channels with a non‐linear structure, filaments were prepared as described above and stained using 33.33 µl mL^−1^ red food color. Filaments were submerged in a 2% (w/v) alginate slurry pre‐cross‐linked with 11.6 mM CaSO_4_ followed by addition of 5 mL 120 mM CaSO_4_ on top. Excess CaSO_4_ was thoroughly rinsed away after 12 hours with MQ. Degradation of the filament was accomplished by addition of 4 mL 0.5 mg mL^−1^ collagenase type 1 solution. In both cases, the remaining degradation products were removed by using a syringe to suction the material. For enhanced visualization, the channel was flushed with a red or blue food color solution in a 30% glucose solution.

### Imaging

Unless otherwise indicated, samples were imaged using a Nikon TiE equipped with a CrEST X‐Light V3 spinning disk module, a Lumencor Celesta laser module, and a Photometrics Prime 95B sCMOS camera using a S Fluor 10x/0.50 objective. Nike Eclipse Ti with a Nikon Intensilight C‐HGFIE module, using either a 4x/0.2 or 10x/0.45 objective, with filter cubes Ex 528–553/Em 590–650 or Ex 625–650/Em 670.

### Statistics

Statistical analysis was performed using GraphPad Prism 10 (San Diego, CA, USA). Data was tested for normality using Shapiro‐Wilk test. Data comparing two groups was analyzed using t‐test. Data comparing more than two groups was analyzed by one‐way ANOVA, followed by Tukey's multiple comparisons test or Kruskal‐Wallis test (*n* > 3). Statistical significance was set at *p* < 0.05 (**** = *p* < 0.001, *** = *p* < 0.001, ** = *p* < 0.01, * = *p* < 0.05).

## Conflict of Interest

The authors declare no conflict of interest.

## Supporting information



Supporting Information

Supplementary Movie 1

Supplementary Movie 2

Supplementary Movie 3

Supplementary Movie 4

Supplementary Movie 5

## Data Availability

The data that support the findings of this study are available from the corresponding author upon reasonable request.
