# Supplementary material for: Printing and Rerouting of Elastic and Protease Responsive Shape Memory Hydrogel Filaments
Source: Adv Healthc Mater. 2025 Jun 20;14(22):2502262. doi: 10.1002/adhm.202502262 (PMC12391619; doi:10.1002/adhm.202502262)
Supplement: Supplementary file 1 — Supporting Information [file ADHM-14-0-s005.pdf]

ADVANCED HEALTHCARE MATERIALS

Supporting Information

for *Adv. Healthcare Mater.*, DOI 10.1002/adhm.202502262

Printing and Rerouting of Elastic and Protease Responsive Shape Memory Hydrogel Filaments

*Philip Lifwergren, Viktoria Schoen, Sajjad Naeimipour, Lalit Khare, Anna Wunder, Hanna Blom, Jose G. Martinez, Pierfrancesco Pagella, Anders Fridberger, Johan Junker and Daniel Aili**

Supplementary information

Printing and Rerouting of Elastic and Protease Responsive Shape Memory Hydrogel Filaments

Philip Lifwergren[1], Viktoria Schoen[1], Sajjad Naeimipour[1], Lalit Khare[1], Anna Wunder[1], Hanna Blom[1], Jose G. Martinez[2], Pierfrancesco Pagella[1], Anders Fridberger[3], Johan Junker[4], Daniel Aili[1,]*

[1] Laboratory of Molecular Materials, Division of Biophysics and Bioengineering, Department of Physics, Chemistry, and Biology, Linköping University, 583 81 Linköping, Sweden.

[2] Sensor and Actuator Systems, Department of Physics, Chemistry and Biology, Linköping University, 581 83 Linköping, Sweden.

[3] Department of Biomedical and Clinical Sciences, Linköping University, 581 85 Linköping, Sweden.

[4] Center for Disaster Medicine and Traumatology, Department of Biomedical and Clinical Sciences, Linköping University, 581 85 Linköping, Sweden.

* Corresponding author: daniel.aili@liu.se

Content

Figure S1. ^{1}H-NMR spectra of HA-BCN in D_2O. 2

Figure S2. Rheology data. 3

Figure S3. Filament diameter as a function of printing pressure and printing speed. 4

Figure S4. Tensile testing of filaments in air. 4

Figure S5. Hydrogel disc swelling and shrinking. 4

Figure S6. Relaxation kinetics of filaments. 5

Figure S7. Rerouting of Free-Floating Suspended Hydrogel Filaments (REFRESH 5

Figure S8. Rheology and viability analysis of bioprinted cell-laden filaments. 6

Figure S9. Embedded perfusable channel structures. 7

References 7

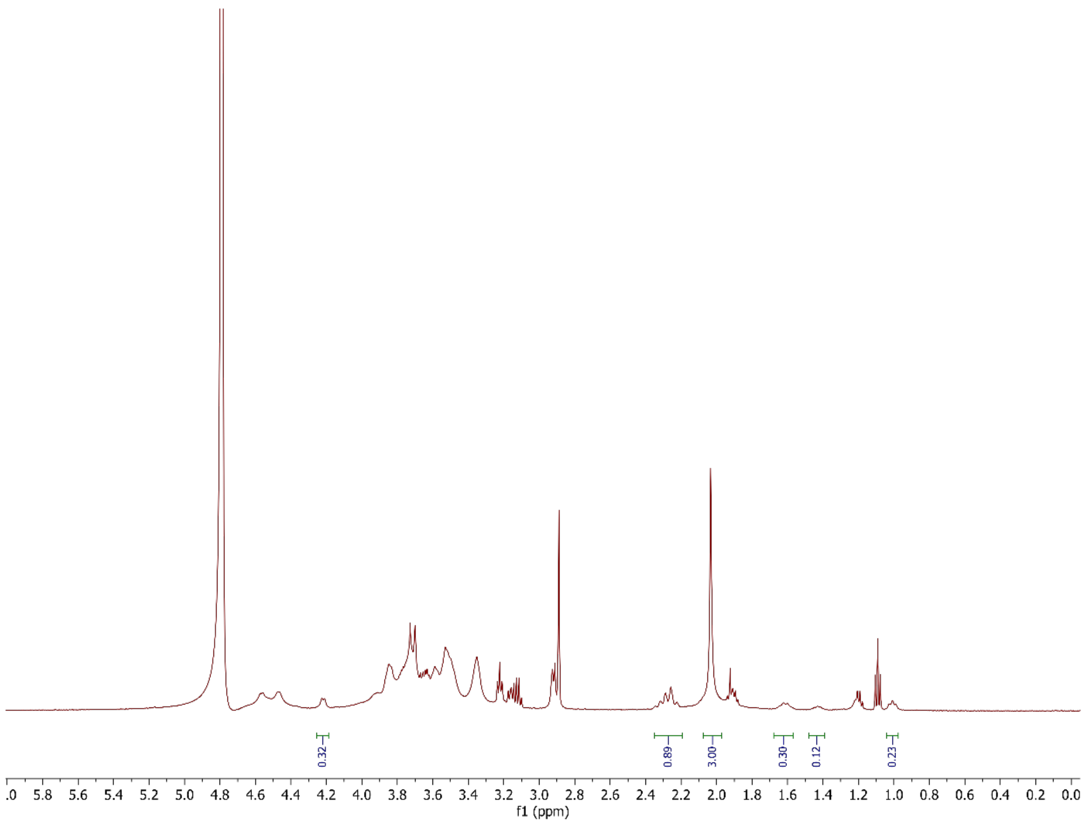

Figure S1. ^{1}H-NMR spectra of HA-BCN in D_2O. The marked peaks were used to calculate the degree of HA functionalized with BCN. The peak from N-acetyl (3H) in HA at 1.95 ppm was used to normalize the remaining BCN peaks[1], allowing for the calculation of a degree of functionalization of 14%.

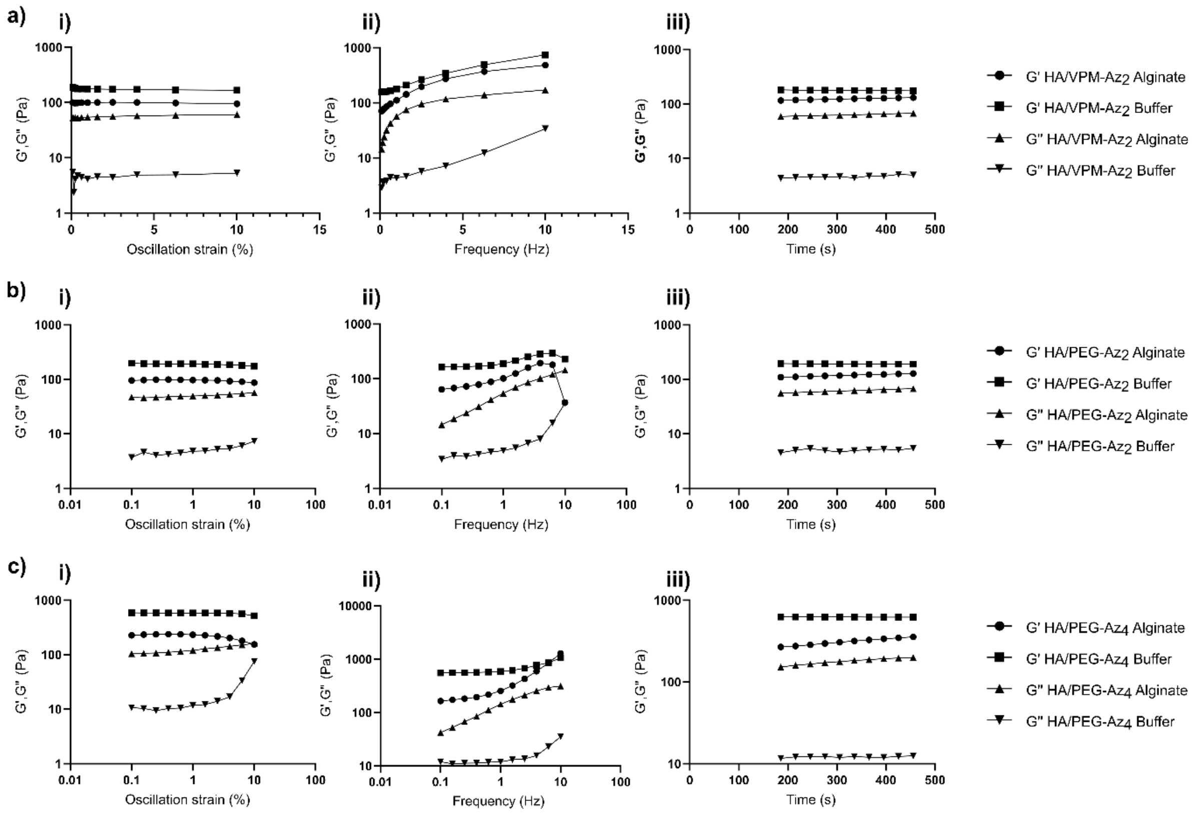

Figure S2. Rheology data. a) HA/VPM-Az_2 hydrogel discs, previously submerged/hydrated in buffer or alginate. i) Amplitude sweep, ii) frequency sweep, and iii) time sweep. b) HA/PEG-Az_2 hydrogel discs, previously submerged/hydrated in buffer or alginate. i) Amplitude sweep, ii) frequency sweep, and iii) time sweep. c) HA/PEG-Az_4 hydrogel discs, previously submerged/hydrated in buffer or alginate. i) Amplitude sweep, ii) frequency sweep, and iii) time sweep.

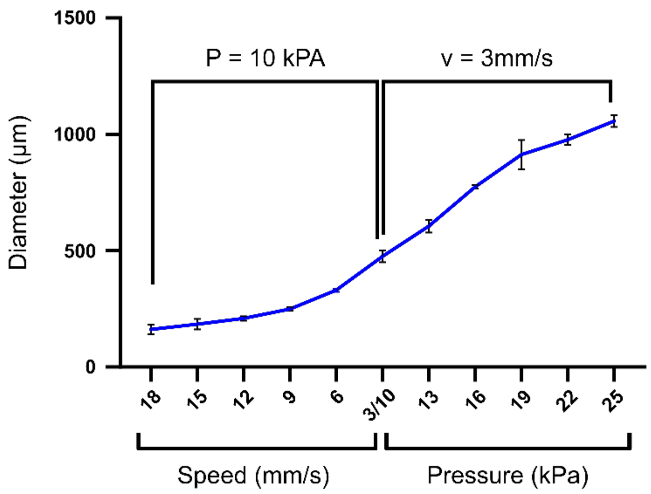

Figure S3. Filament diameter as a function of printing pressure and printing speed.

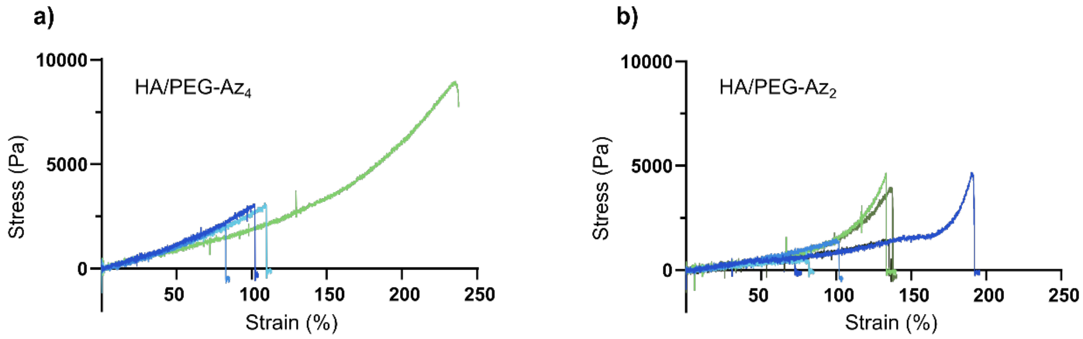

Figure S4. Tensile testing of filaments in air. a) HA/PEG-Az4. b) HA/PEG-Az2.

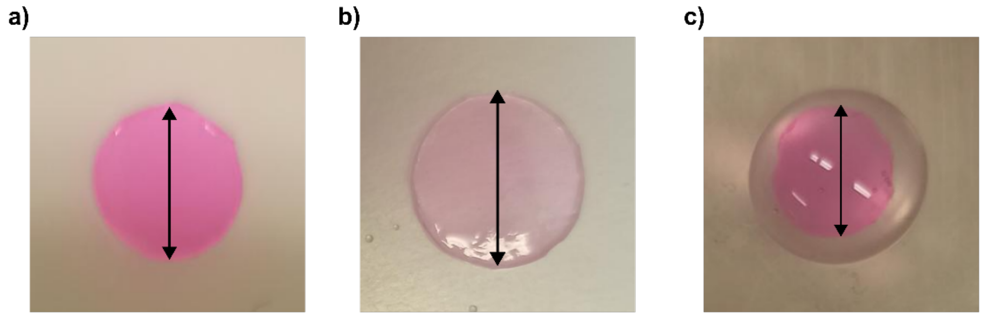

Figure S5. Hydrogel disc swelling and shrinking. a) Control hydrogel disc with a diameter of 8 mm. b) Hydrogel disc swelled in buffer to a diameter greater than 8 mm. c) Hydrogel disc covered with alginate shrunk to a diameter less than 8 mm.

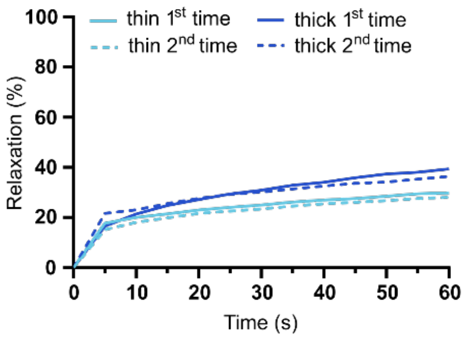

Figure S6. Relaxation kinetics of filaments. Thin and thick filaments after the first stretching compared to when stretched out a second time.

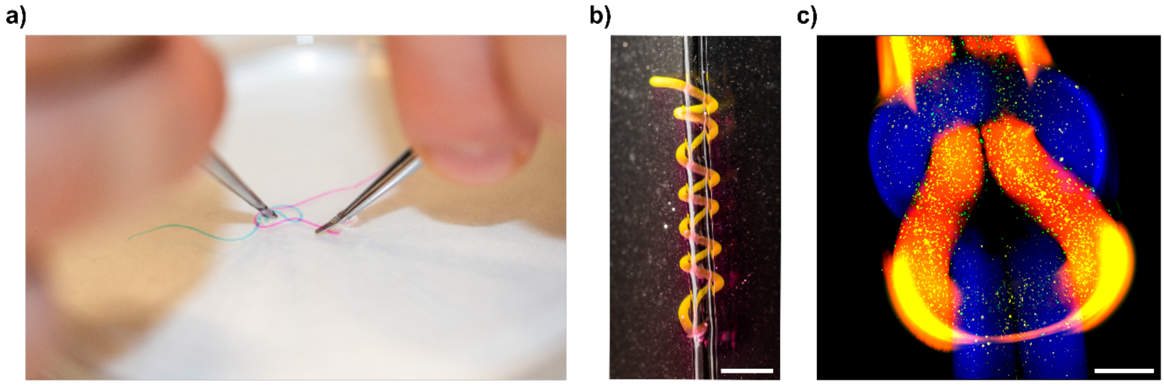

Figure S7. Rerouting of Free-Floating Suspended Hydrogel Filaments (REFRESH). a) Manually tying a knot by joining two hydrogel filaments. b) Filament winded around a glass cylinder with a diameter of 1 mm. c) Coculture of fibroblasts within the red filament and MDA-MB-231 within the blue filament. Scale bars: 5 mm.

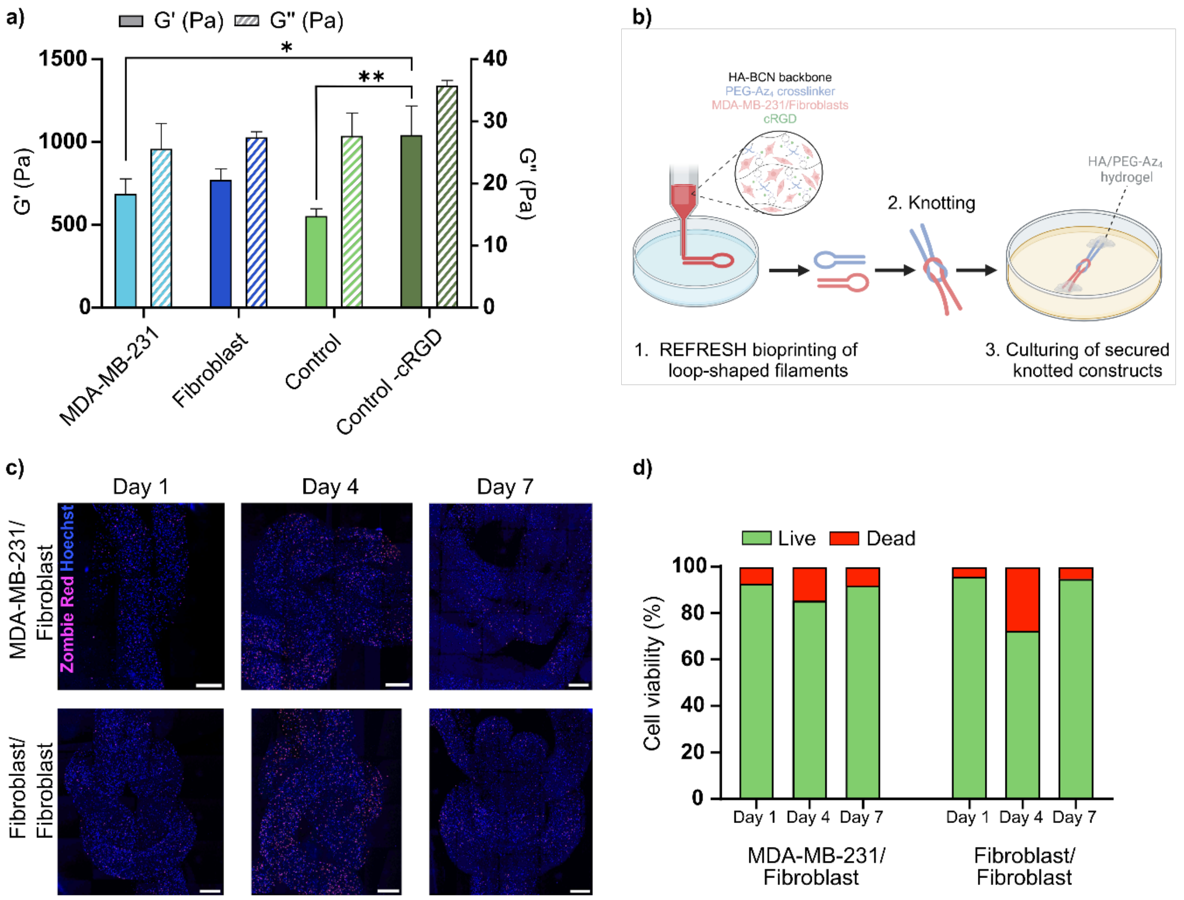

Figure S8. Rheology and viability analysis of bioprinted cell-laden filaments. a) Storage modulus (G′) and loss modulus (G″) of MDA-MB-231 laden HA/PEG-Az_4 + cRGD hydrogels, fibroblast-laden HA/PEG-Az_4 + cRGD hydrogels, and cell-free HA/PEG-Az_4 hydrogels ± cRGD. b) Illustration of the process for fabrication and culture knotted filaments. c) Confocal fluorescence images of knots of filaments with MDA-MB-231 and fibroblast cells stained with Hoechst 33342 (blue) for nuclei and Zombie Red (pink) for non-viable cells. d) Quantification of cell viability (%) in cell-laden filament knots of filaments with MDA-MB-231 and fibroblast cells. Scale bars: 500 µm.

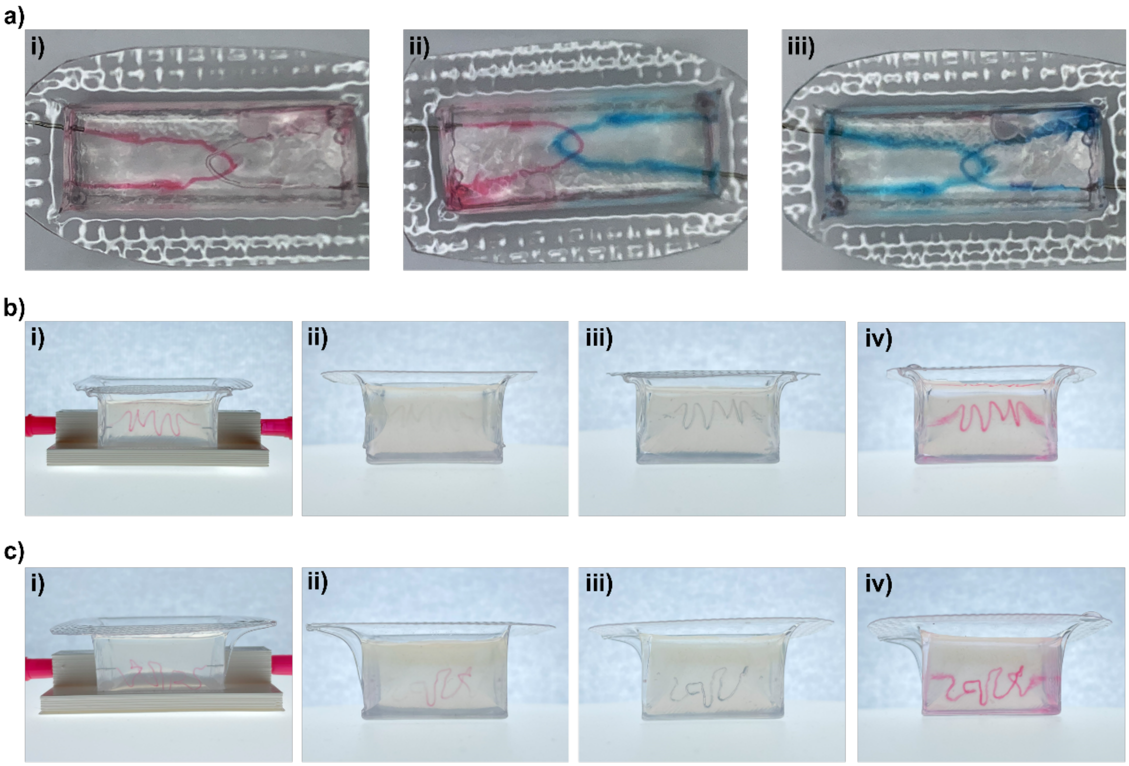

Figure S9. Embedded perfusable channel structures. a) REFRESH filaments were degraded to form channels that intertwined with each other: i) Perfusion of the left channel with a red buffer, while the right channel is empty, ii) a blue buffer flows through the right channel, iii) the red buffer is replaced by a blue buffer. b,c) i) Embedded REFRESH filament in supporting gel matrix post $CaSO_4$ crosslinking. ii) Remaining filament after overnight col-I degradation. iii) Hollow channel after clearance of residual material. iv) Perfusion of the created channel with a red buffer.

References

[1] J. Dommerholt, S. Schmidt, R. Temming, L. J. A. Hendriks, F. P. J. T. Rutjes, J. C. M. Van Hest, D. J. Lefeber, P. Friedl, F. L. Van Delft, *Angew. Chemie - Int. Ed.* **2010**, *49*, 9422.
